# Epidemiology and outcome of influenza‐associated infections among hospitalized patients with acute respiratory infections, Egypt national surveillance system, 2016‐2019

**DOI:** 10.1111/irv.12867

**Published:** 2021-05-07

**Authors:** Manal Fahim, Basma AbdElGawad, Hossam Hassan, Amel Naguib, ElSabbah Ahmed, Salma Afifi, Hanaa Abu ElSood, Amira Mohsen

**Affiliations:** ^1^ Department of Epidemiology and Surveillance, Preventive Sector Ministry of Health and Population Cairo Egypt; ^2^ Central Public Health Laboratory Ministry of Health and Population Cairo Egypt; ^3^ Ministry of Health and Population Consultant Cairo Egypt; ^4^ Egypt Country Office World Health Organization Cairo Egypt

**Keywords:** epidemiology, hospitalization, influenza, public health surveillance

## Abstract

**Introduction:**

Egypt has established different types of surveillance systems to monitor influenza activities, early detect outbreaks, and tailor efficient prevention and control strategies. This is the first study to describe epidemiology and outcome of influenza‐associated infections among hospitalized patients using the National Electronic Disease Surveillance System (NEDSS) data, 2016‐2019.

**Methods:**

Data reported from 284 hospitals all over Egypt were extracted from the NEDSS. Data of hospitalized patients with Acute Respiratory Infections (ARI), 2016‐2019, were included in the analysis. Laboratory testing for influenza by RT‐PCR according to US CDC testing protocol was used to confirm influenza type and subtype.

**Results:**

Overall 46 417 patients hospitalized with ARI were identified, their mean age was 30.9 ± 26 and 52.9% were males. Among 41 512 (89.4%) laboratory‐tested patients, 7167 (17.3%) were positive for one or more types of influenza viruses. Influenza viruses circulated in all ages and throughout the year, with higher rates in winter, late childhood, and middle ages. Mortality from influenza was significantly higher than other causes of ARIs (5.0% vs 3.8%, *P* < .001), and it was associated with older ages, December‐May, delay in hospital admission, residence in urban and frontier governorates and infection with A/H1N1 virus. The distribution of influenza subtype by time shows alternate pattern between A/H1N1 and H3N2, each subtype peaks every other year with a high peak of A/H1N1 in 2016.

**Conclusions:**

The national Egyptian surveillance succeeded to describe the epidemiology of hospitalized patients with ARIs and influenza in Egypt over time. Surveillance with strain‐specific laboratory testing and annual assessment of associated severity might be useful to guide influenza prevention and control strategies including vaccination and case management.

## 1 INTRODUCTION

Influenza‐associated infections are a major contributor to morbidity and mortality worldwide. The World Health Organization (WHO) has indicated that about 3‐5 million cases and 290 000‐650 000 deaths occur annually from seasonal influenza epidemics.^1^ Influenza remains a global public health priority because of the threat of a new global pandemic.

Data on influenza epidemiology and burden are available from developed countries, yet little is known about the disease in low‐income and tropical countries. In response to this gap, WHO led an initiative to establish virological surveillance systems as part of national pandemic preparedness efforts.^2^ Although those systems have produced substantial data on the epidemiology and impact of influenza, yet lack of quality data on disease severity and outcomes, and high‐risk groups remains a problem.^3^

In Egypt, the Ministry of Health and Population (MoHP) started monitoring trends in the incidence of influenza serotypes through influenza virologic ILI sentinel surveillance since 1999. In 2002, a laboratory‐based National Electronic Disease Surveillance System (NEDSS) targeting 40 infectious diseases was established as a desktop application. NEDSS was upgraded to online web‐based application in 2012 parallel to paper‐based reporting. In 2016, online reporting completely replaced paper‐based reporting after ensuring good performance indicators of the online reporting system. Using NEDSS online, all governmental health facilities including primary health units, chest, and general and fever hospitals are required to report infectious diseases to the Department of Epidemiology and Surveillance (DES) at MoHP within 48 hours.

In response to the 2009 A/H1N1 pandemic influenza, a memorandum was issued by MoHP requiring all governmental hospitals to report any patient with Acute Respiratory Infection (ARI) attending outpatient clinic or admitted to governmental hospital through NEDSS within 48 hours. In addition, all other types of hospitals including teaching, health insurance, university, and private hospitals were invited to voluntarily report aggregate data on ARI on a weekly basis to MoHP.

This report presents the results of the national laboratory‐based surveillance for hospitalized patients with ARI in 284 hospitals all over Egypt, 2016‐2019. The study aims at describing the epidemiology and exploring severity and mortality of influenza‐associated infections among hospitalized ARI patients to identify target groups for influenza prevention and control strategies.

## 2 METHODS

### 2.1 Study subjects, patients' enrollment, and data collection

Subjects are all patients hospitalized with history or measured fever of ≥38°C and cough within the last 10 days prior to disease onset and all those who are clinically or radiologically diagnosed having pneumonia, 2016‐2019. The WHO case definition for Severe Acute Respiratory Infections (SARI) and/ or physician clinical judgment was used for patient enrollment. Enrolled patients were interviewed using the surveillance standard form. All patients are requested to provide oropharyngeal and nasopharyngeal swabs for influenza testing by RT‐PCR to identify the influenza type and subtype using the CDC protocol.^4^ Specimens are placed in Viral Transport Media (VTM) and kept in an icebox, before being transferred within 24 hours to the nearest regional laboratory or Central Public Health Laboratory (CPHL) in Cairo for testing. Patients are interviewed by the hospital surveillance officer using a standardized questionnaire that includes patient demographic data, signs and symptoms and ARI risk factors. Data are entered using the online NEDSS application.

### 2.2 Data extraction, validation, and analysis

National data for patients hospitalized with ARI were extracted from the NEDSS database from 2016‐2019. All patients hospitalized with ARIs during this period were included in the study, while patients attended outpatient clinic were excluded. Data were completed and validated by surveillance officers at each reporting site using facility records. Descriptive data analysis was performed for available data including demographics, influenza type and subtype, history of comorbidities, and patient outcome using SPSS ver. 25. Influenza‐associated infection (FAI) rate was calculated as the proportion of specimens positive for influenza out of the total specimens tested. Seasonality was described by calculating rate of influenza positivity by month, with the highest positivity rate considered as peak.

Difference between influenza‐positive and influenza‐negative hospitalized patients was examined using bivariate analysis. Influenza mortality risk factors were evaluated by comparing patients with influenza‐associated infections who died at hospital to those discharged alive. Risk factors included (age, gender, days from symptom onset to hospitalization, chronic conditions, year, season, region, and influenza subtype). Pearson's chi‐square was used to evaluate the difference between categorical variables and *t* test for continuous variables, with statistical significance set at *P* value <.05. Linear regression was performed to examine the relation between influenza A/H1N1 and influenza mortality by region and year's season.

## 3 RESULTS

Out of 1 123 921 communicable diseases reported to NEDSS program from January 2016‐December 2019; there were 204 786 patients who attended outpatient clinics or admitted to hospitals with ARIs representing 18.2% of all reported communicable diseases patients and ranked as the second notifiable disease following diarrheal diseases. From the 204 786 ARI reported patients; 46 417 were hospitalized giving ARI hospitalization rate of 22.7%.

### 3.1 Epidemiologic characteristics of influenza‐positive and influenza‐negative hospitalized patients

The mean age of the 46 417 hospitalized ARI patients was 30.9 ± 26 years, ranging from 1 day to 99 years with almost 1/3 of them (29.8%) in the middle age‐group (15‐49 years) and 52.9% were males. The highest number of patients 13 729 (29.6%) were reported in 2019, with 64.2% of cases occurred between December and May. More than half of patients (51.8%) were from Lower Egypt, while 25.5% from Upper Egypt, 20.3% urban governorates, and 2.4% were from frontier governorates. Overall 1754 (3.8%) patients with ARIs died during hospitalization (Table 1).

**TABLE 1:** Demographic characteristics of influenza‐positive and influenza‐negative hospitalized patients with acute respiratory infection (ARI)—Egypt, 2016‐2019

	All hospitalized ARI patients				Influenza positive		Influenza negative		OR	95% CI	*P* Value
	Total no.	Of total	No. swabbed	% Swabbed	No	%	No	%			
All cases	46 417	100.0	41 512	89.4	7164	17.3	34 348	82.7	NA	NA	NA
Year											
2016	10 826	23.3	10 702	98.9	2742	25.6	7960	74.4	Ref	Ref	Ref
2017	10 278	22.1	8549	83.2	780	9.1	7769	90.9	0.3	0.2‐0.4	<.001
2018	11 584	25.0	10 501	90.7	1894	18.0	8607	82.0	0.6	0.5‐0.8	<.001
2019	13 729	29.6	11 760	85.7	1748	14.9	10 012	85.1	0.5	0.47‐0.54	<.001
Season											
June‐August	6669	14.4	6098	91.4	414	6.8	5684	93.2	Ref	Ref	Ref
September‐November	9941	21.4	9084	91.4	1776	19.6	7308	80.4	3.3	3.0‐3.7	<.001
December‐February	17 187	37.0	14 909	86.7	3342	22.4	11 567	77.6	4.0	3.6‐4.4	<.001
March‐May	12 620	27.2	11 421	90.5	1632	14.3	9789	85.7	2.2	2.0‐2.6	<.001
Age‐group years											
<2	8061	17.4	7187	89.2	522	7.3	6665	92.7	Ref	Ref	Ref
2‐4	5320	11.5	4492	84.4	834	18.6	3658	81.4	2.9	2.6‐3.3	<.001
5‐14	4427	9.5	3607	81.5	839	23.3	2768	76.7	3.9	3.4‐4.4	<.001
15‐49	13 852	29.8	12 467	90.0	2578	20.7	9889	79.3	3.3	3.0‐3.7	<.001
50‐64	9085	19.6	8464	93.2	1638	19.4	6826	80.6	3.0	2.8‐3.4	<.001
≥65	5672	12.2	5295	93.4	753	14.2	4542	85.8	2.1	1.9‐2.4	<.001
Gender											
Males	24 565	52.9	22 156	92.2	3679	16.6	18 477	83.4	1.1	1.0‐1.2	<.001
Females	21 852	47.1	19 356	88.6	3485	18.0	15 871	82.0			
Region											
Upper Egypt	11 856	25.5	11 304	95.3	1707	15.1	9597	84.9	Ref	Ref	Ref
Lower Egypt	24 049	51.8	22 809	94.8	4075	17.9	18 734	82.1	1.2	1.1‐1.3	<.001
Urban governorates	9421	20.3	6409	68.0	1105	17.2	5304	82.8	1.2	1.1‐1.3	<.001
Frontier governorates	1091	2.4	990	90.7	277	28.0	713	72.0	2.2	1.9‐2.5	<.001
Outcome											
Died during hospitalization	1763	3.8	1666	94.5	357	21.4	1309	78.6	1.3	1.2‐1.5	<.001
Discharged	44 654	96.2	39 846	89.2	6807	17.1	33 039	82.9			

Among 41 512 (89.4%) laboratory‐tested patients, 7167 (17.3%) were positive for one or more types of influenza viruses. FAI rate was highest in 2016 (25.6%) followed by 2018 and 2019 (18.0% and 14.9%, respectively) compared with 2017 (9.1%, *P* < .001). It was highest between December‐February (22.4%), high in September‐November and March‐May periods (19.4, 14.3%, respectively) compared with June‐August (6.8%), *P* < .001. More than 1/3 (36.0%) of influenza cases fall in the middle age‐group (15‐49 years), and FAI rate was significantly higher in females than males (18.0% vs 16.6%, *P* < .001), frontier, lower Egypt, and urban governorates than Upper Egypt (28.0, 17.9%, 17.2% vs 15.1%) and in patients who died during hospitalization (21.4%) compared with those discharged alive (17.1%, *P* < .001). (Table 1).

### 3.2 Comparison between influenza types/subtypes

Among the 7164 influenza‐positive infections, A/H1N1 was the cause in 2990 (41.7%) patients, 1773 (24.1%) A/H3N2, 2357 (32.9%) Flu‐B and 44 (0.6%) were mixed two types of influenza infection. A/H1N1 was the dominant virus in 2016 representing 51.8% of influenza‐associated infections, while Flu‐B dominated in 2017 (79.3%). In 2018, H3N2 and A/H1N1 co‐dominated over Flu‐B representing (38.6% and 37.3%) and in 2019 A/H1N1 and Flu‐B co‐dominated over H3N2 representing (48.0% and 40.8%) of cases. (Table 2).

**TABLE 2:** Comparison between influenza types/subtypes as a cause of ARI, National Egyptian Surveillance, 2016‐2019

	Total^a^ number	H1N1		H3N2		Flu‐B		*P* Value
		No	%	No	%	No	%	
All cases	7120	2990	42.0	1773	24.9	2357	33.1	<.001
Age‐group years								
<2	520	226	43.5	110	21.2	184	35.4	.115
2‐4	828	328	39.6	191	23.1	309	37.3	.023
5‐14	836	232	27.8	166	19.9	438	52.4	<.001
15‐49	2569	1130	44.0	621	24.2	818	31.8	.037
50‐64	1624	800	49.3	427	26.3	397	24.4	<.001
≥65	743	274	36.9	258	34.7	211	28.4	<.001
Gender								
Males	3654	1532	41.9	923	25.3	1199	32.8	.746
Females	3466	1458	42.1	850	24.5	1158	33.4	
Symptoms onset to hospitalization (mean ±SD)	4.8 ± 4.0	5.1 ± 5.0		4.7 ± 3.9		4.6 ± 3.5		<.001
Outcome								
Died	353	245	69.4	58	16.0	50	14.2	<.001
Discharged	6767	2745	40.6	1715	25.3	2307	34.1	
Year								
2016	2727	1413	51.8	733	26.9	581	21.3	<.001
2017	777	44	5.7	117	15.1	616	79.3	<.001
2018	1886	703	37.3	728	38.6	455	24.1	<.001
2019	1730	830	48.0	195	11.3	705	40.8	<.001
Season								
June‐August	399	35	8.8	105	26.3	259	64.9	<.001
September‐November	1763	451	25.6	832	47.2	480	27.2	<.001
December‐February	3330	1968	59.1	656	19.7	706	21.2	<.001
March‐May	1628	536	32.9	180	11.1	912	56.0	<.001
Region								
Lower Egypt	4064	1542	37.9	1093	26.9	1429	35.2	<.001
Upper Egypt	1688	670	39.7	431	25.5	587	34.8	.082
Urban governorates	1092	600	54.9	216	19.8	276	25.3	<.001
Frontier governorates	276	178	64.5	33	12.0	65	23.6	<.001

^a^ Cases with co‐infections are not presented.

**FIGURE 1:**
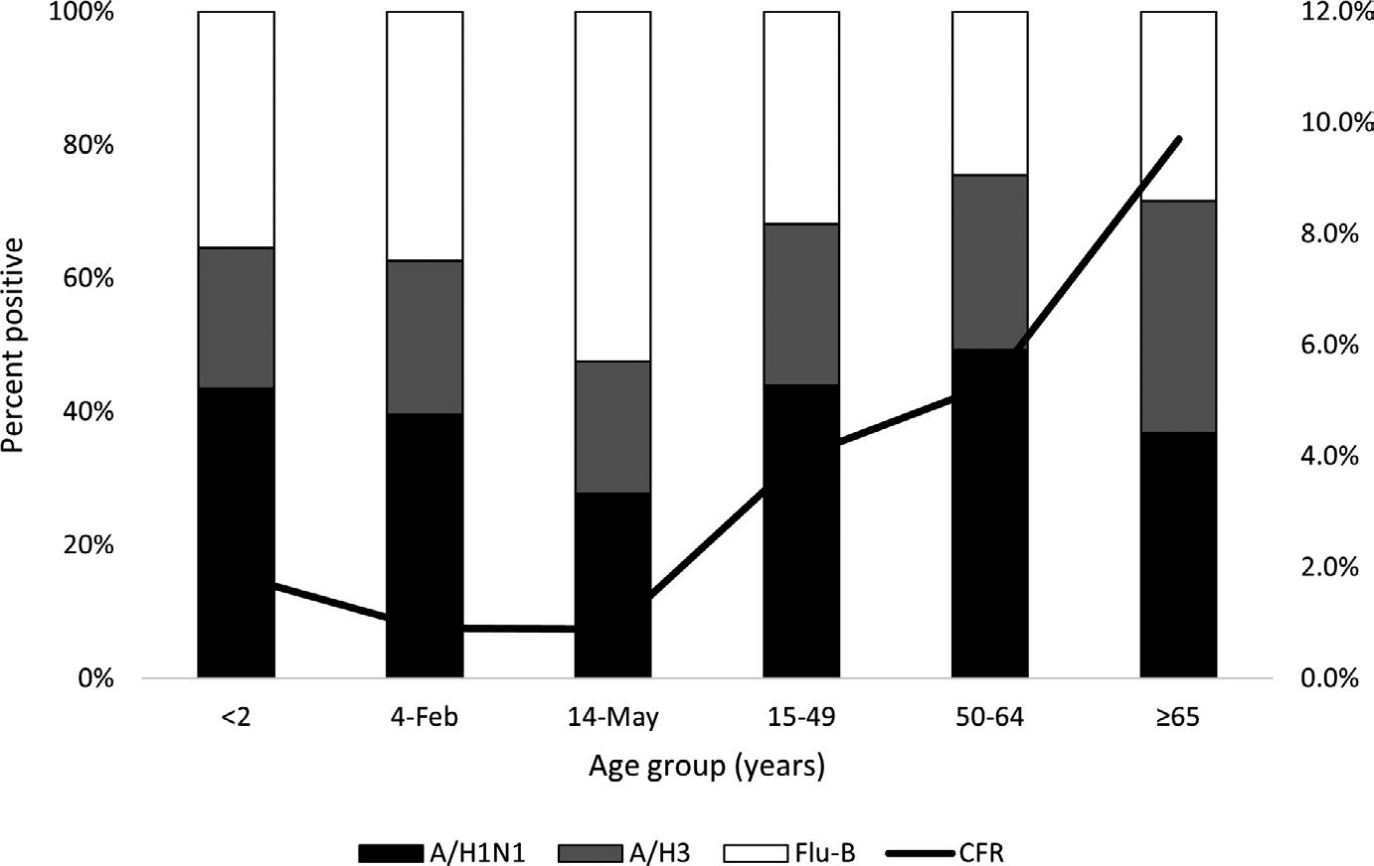
Distribution of Influenza subtypes and case fatality rate (CFR) among hospitalized ARI patients by age‐groups, National Egyptian Surveillance, 2016‐2019

Influenza A/H1N1 was the main cause of influenza‐associated infections among <5 years age‐group, and middle age‐groups (15‐64), while in older ages ≥65 A/H1N1 and A/H3N2 were the causes and Flu‐B was the main cause in late childhood (5‐14) years. Figure 1. Most of the infections with A/H1N1 occur in December‐February months representing 59.1% of influenza admissions in this period, while most cases with A/H3N2 occurs in September‐December causing 47.2% and most Flu‐B cases occur March‐May causing 56.0% of influenza admissions in the corresponding season (Table 2).

A/H1N1 represented the main subtype of influenza in the urban and frontier governorates (54.9 and 64.5%) of influenza hospitalized patients, and A/H1N1 and Flu‐B were reported from Upper and Lower Egypt more than A/H3N2. Most of deaths from influenza during hospitalization (69.4%) were caused by A/H1N1; patients with A/H1N1 took longer time before hospital admission than H3N2 and Flu‐B patients (mean duration 5.1 ± 5.0 vs 4.7 ± 3.9 and 4.6 ± 3.5, *P* < .001) (Table 2).

**FIGURE 2:**
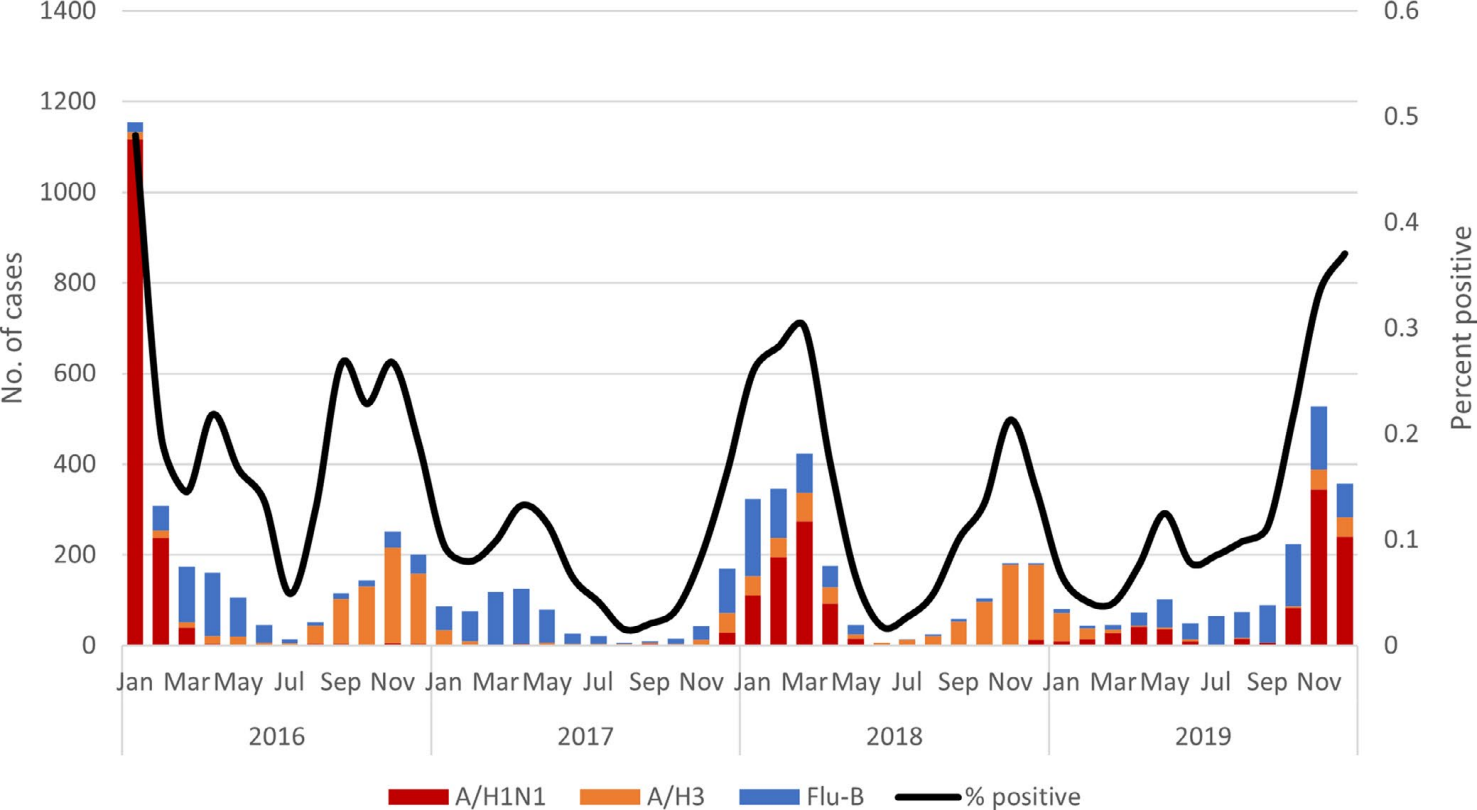
Distribution of three influenza subtypes by month as a cause of patients hospitalized with ARI, National Egyptian Surveillance, 2016‐2019

The distribution of influenza subtype by time shows alternate pattern between A/H1N1 and H3N2, each subtype peaks every other year with a high peak of A/H1N1 in 2016. There was an unusual peak of Flu‐B in 2017 which caused next influenza season to start late in November (Figure 2).

### 3.3 Influenza mortality

The case fatality rate was highest in old age‐groups 50‐64 and ≥65 years (7.8 and 11.3%, respectively), while it was lowest in late childhood 5‐14 years (0.7%). Mortality was significantly higher December‐February compared with June‐August (7.0% vs 1.0, *P* < .001), in urban and frontier governorates than Upper Egypt (8.6%, 7.6% vs 3.7%), in years 2016 and 2019 compared with 2017 (5.9%, 5.5% vs 1.4%, *P* < .001). Risk factors for death from influenza included diagnosis of pneumonia and being late for seeking medical care (8.8 vs 2.4, *P* < .001 and 6.9 ± 6.3 vs 4.8 ± 4.2, *P* < .001). By subtype, it was highest in A/H1N1 compared with H3N2 and Flu‐B (8.2 vs 3.3 and 2.1%, respectively, *P* < .001). Mortality was associated with diagnosis of pneumonia (Table 3) Most of the patients (77.3%) with confirmed influenza who died during hospitalization had history of one or more chronic disease including 23.1% diabetics, 9.6% had chronic obstructive pulmonary disease, 5.8% cardiovascular disease, 4.6% chronic hepatic disease, and 6.5% others (renal, neurologic, malignancy, or autoimmune disease).

**TABLE 3:** Risk factors for influenza mortality among patients hospitalized with ARI, National Egyptian Surveillance, 2016‐2019

	Died during hospitalization (n = 353)		Discharged alive (n = 6767)		OR	95% CI	*P* Value
	No	%	No	%			
Age‐group years							
<2	9	1.7	511	98.3	Ref	Ref	Ref
2‐4	10	1.2	818	98.8	0.7	0.3‐1.8	.437
5‐14	6	0.7	830	99.3	0.4	0.1‐1.2	.073
15‐49	118	4.6	2451	95.4	2.7	1.4‐5.8	.001
50‐64	126	7.8	1498	92.2	4.8	2.5‐10.1	<.001
≥65	84	11.3	659	88.7	7.2	3.7‐15.4	<.001
Gender							
Males	188	5.1	3466	94.9	1.1	0.9‐1.3	.455
Females	165	4.8	3301	95.2			
Influenza type/subtype^a^							
Flu‐B	50	2.1	2307	97.9	Ref	Ref	Ref
A/H3	58	3.3	1715	96.7	1.6	1.1‐2.3	.01
A/H1N1	245	8.2	2745	91.8	4.1	3.0‐5.7	<.001
Season							
June‐August	4	1.0	395	99.0	Ref	Ref	Ref
September‐November	59	3.3	1704	96.7	3.4	1.3‐11.1	<.01
December‐February	232	7.0	3098	93.0	7.4	3.0‐23.6	<.001
March‐May	58	3.6	1570	96.4	3.6	1.4‐11.9	<.01
Region							
Upper Egypt	63	3.7	1625	96.3	Ref	Ref	Ref
Lower Egypt	175	4.3	3889	95.7	1.2	0.9‐1.6	1.161
Frontier governorates	21	7.6	255	92.4	2.1	1.3‐3.5	<.01
Urban governorates	94	8.6	998	91.4	2.4	1.8‐3.4	<.001
Year							
2016	161	5.9	2566	94.1	Ref	Ref	Ref
2017	11	1.4	766	98.6	0.2	0.1‐0.4	<.001
2018	86	4.6	1800	95.4	0.8	0.6‐1.0	.023
2019	95	5.5	1635	94.5	0.9	0.7‐1.2	.283
Mean duration onset‐ hospital admission ± SD	6.9 ± 6.3			4.8 ± 4.2			<.001
Pneumonia	138	8.8	1425	91.2	2.4	2.0‐3.0	<.001
No pneumonia	215	3.9	5342	96.1			
Chronic diseases^b^	170	77.3	NA	NA	‐	‐	**‐**
Diabetes	60	23.1	NA	NA	‐	‐	**‐**
COPD	25	9.6	NA	NA	‐	‐	**‐**
Cardiovascular disease	15	5.8	NA	NA	‐	‐	**‐**
Hepatic	12	4.6	NA	NA	‐	‐	**‐**
Renal	10	3.8	NA	NA	‐	‐	**‐**
Malignancy	4	1.5	NA	NA	‐	‐	**‐**
Pregnancy^c^	10	7.8	NA	NA	‐	‐	**‐**
Others^d^	17	6.5	NA	NA	‐	‐	**‐**
>one chronic disease	17	6.5	NA	NA	‐	‐	**‐**

^a^ Cases with co‐infection were excluded from the analysis (N = 7120).

^b^ Information on history of chronic disease is available only for the 260 patients died during hospitalization with influenza.

^c^ Out of 128 females died with influenza.

^d^ Other include Hematologic, neurologic, and autoimmune disease and smoking.

**FIGURE 3:**
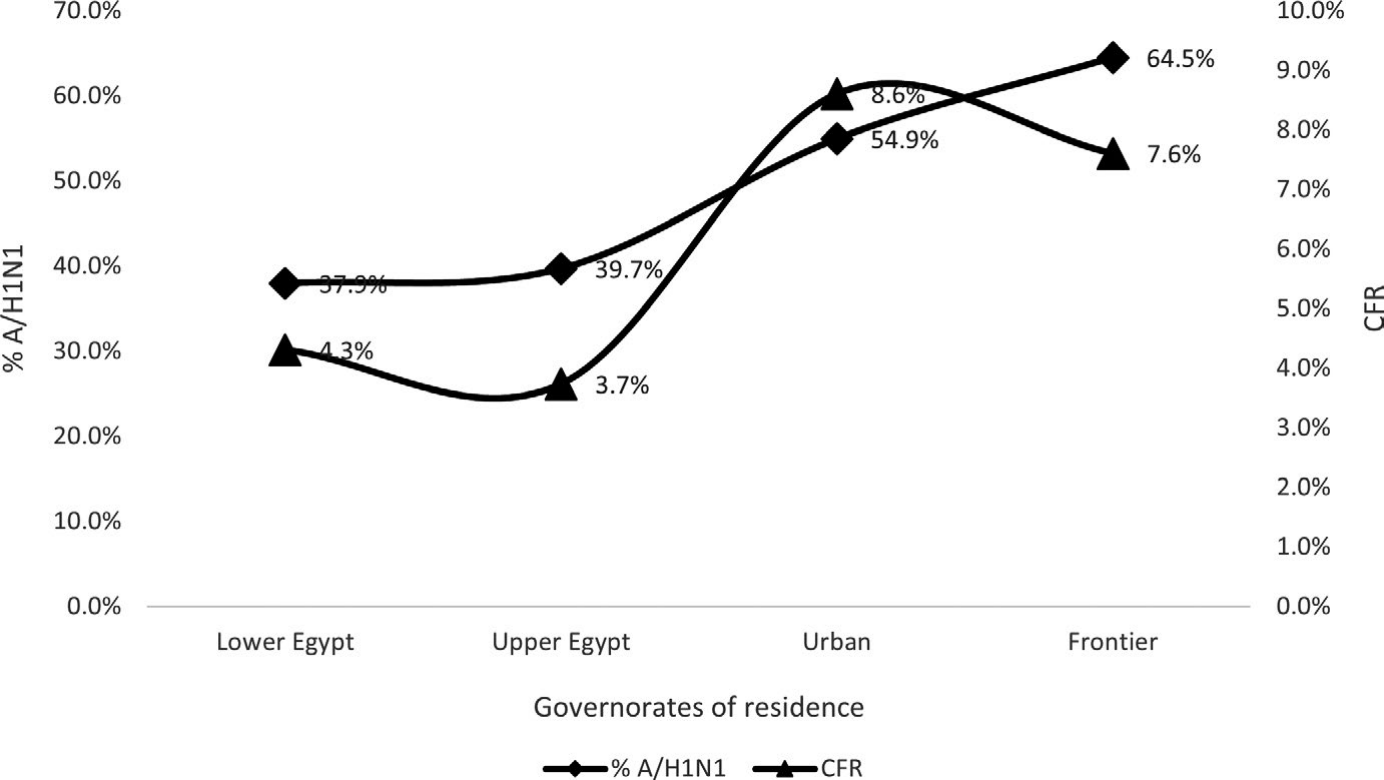
Relation between case fatality rate and percent of influenza A/H1N1 in patients hospitalized with ARI in different Egyptian regions, National Egyptian Surveillance, 2016‐2019

**FIGURE 4:**
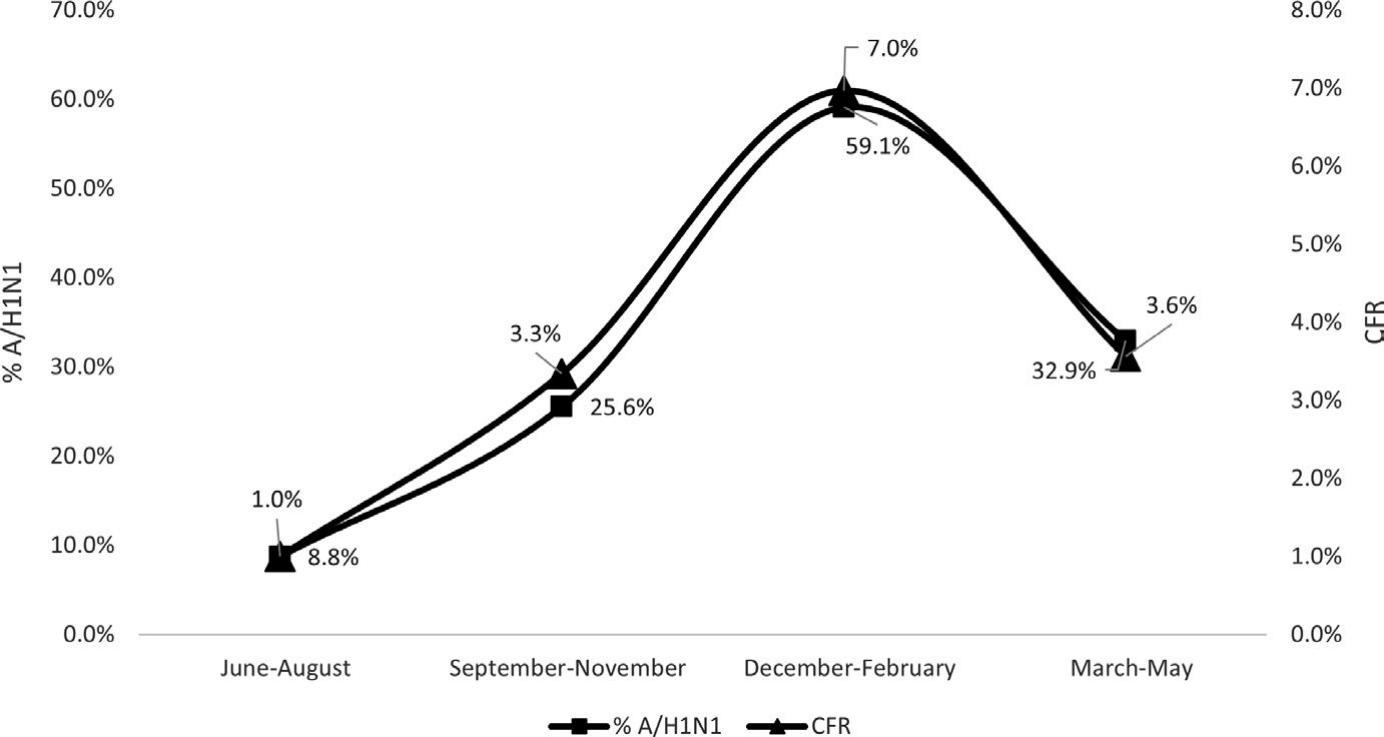
Relation between case fatality rate and percent of influenza A/H1N1 by season in patients hospitalized with ARI, National Egyptian Surveillance, 2016‐2019

A positive correlation was found between percent of A/H1N1 subtype and CFR from influenza among hospitalized patients by Egyptian regions and season of the year (*R* ^2^=0.75 and 0.90, respectively). The variation of CFR from influenza among hospitalized patients by Egyptian regions and season of the year is linked to percent of A/H1N1 infections in those groups. (Figures 3 and 4).

## 4 DISCUSSION

Acute Respiratory Infection (ARI) is one of the top causes of morbidity and mortality worldwide.^5^ Of 13 studies conducted at Eastern Mediterranean Region (EMR) to calculate the proportionate morbidity, 11 ranked ARIs as having the highest or second‐highest proportionate morbidity among other illnesses.^6^ This study proved that ARI is one of the main causes of morbidity in Egypt, where it ranked them as the second notifiable infectious disease following diarrheal diseases.

Most of the studies published from EMR and all the studies from Egypt describing the epidemiology and burden of ARI were conducted in sentinel sites or in one district.^6^, ^7^, ^8^ This is the first study to present laboratory‐based national data on Egyptian populations hospitalized with ARIs reported from 284 hospitals all over the different regions across the country.

The overall rate of FAI in this study (17.3%) was comparable to results of a study conducted in one district in Lower Egypt, 2013 (19.0%) ^7^ and slightly lower than results of a study conducted in 15 sub‐Saharan countries in 2016 (21.7%).^9^ Although the overall rate is comparable to previous studies from Egypt and the region, yet it differs by year with a peak in 2016 compared with low rate in 2017 and moderate rate in 2018 and 2019, in line with a study from Oman reported different influenza positivity rate by year.^10^ This finding emphasizes the importance of surveillance in monitoring influenza activity to develop and update the influenza preparedness plans.

In regions closer to the equator, influenza may occur throughout the year and some tropical countries may experience two peaks of activity annually.^11^, ^12^ This study proved that transmission of influenza viruses occurs all over the year with two peaks every year, a finding reported in other studies from Egypt and the region.^7^, ^13^ This should be considered in the planning for vaccination production and supply and in developing the preparedness plans.

The finding that rate of FAI differs by region within Egypt was also reported by other studies from Iran.^14^ This finding is most probably due to the variability of surveillance performance in the different country regions. The high rate in urban governorate identified in this study could be related to better application of case definition and laboratory testing, while under reporting of influenza‐negative cases in frontier governorates could be the reason for high influenza‐associated infection rates (FAI). Other possible causes could be seasonal variability of different influenza subtypes, different levels of immunity among these populations, or different levels of applying prevention and control measures for the at‐risk groups.

A(H1N1) virus caused more than 40% of all FAIs, dominated in 2016 and co‐dominated with influenza A(H3N2) in 2018 and with Flu‐B in 2019, while Flu‐B dominated in 2017. This is in line with what has been reported in temperate countries of Northern and Southern hemispheres for the same period.^15^ Influenza viruses monitoring by subtype is critical for tracking the movement of viruses globally, regionally and nationally and interpreting the epidemiological data.

In this study, influenza was the cause of ARI in all ages >2 years, by subtype rate was higher in middle ages for A/H1N1, while it was higher very old ages for H3N2 and for Flu‐B it was high in younger ages. This was in line with a study in the United States,^16^ while contrasts studies from Egypt and the region where different affected age‐groups were identified for each subtype.^6^, ^7^, ^13^ The variability between different studies could be explained by the difference in case definition used, dominant influenza subtype, seasonality, and severity of the circulating strains. This finding could help in diagnosis of influenza subtype using patient age if laboratory testing unavailable.

The high mortality rate among FAIs identified in this study among older age‐groups is also reported by many studies which indicated that risk of fatality from influenza increases with age.^16^, ^17^, ^18^

Similar to previous studies that reported high mortality due to influenza among hospitalized patients and higher CFRs for A/H1N1,^8^ this study reported high influenza mortality rate and indicated that >¾ of all deaths from influenza was due to A/H1N1. This highlights the value of laboratory influenza testing by type and subtype especially for severe cases and the continuous revision of influenza management guidelines based on surveillance results.

The high CFR and the positive correlation between percent of A/H1N1 and fatal outcome by region and season reported in this study was in line with many other studies that reported high pathogenicity and mortality rates from A/H1N1.^19^, ^20^ The cause of death from A/H1N1 is usually the higher possibility of severe involvement of the lungs leading to respiratory failure.^21^ Another reason for high mortality from A/H1N1 suggested from this study is the delay in hospitalization compared with other subtypes. Studies indicated that the secondary bacterial pneumonia which may complicate influenza A/H1N1 occurs in a biphasic pattern of signs and symptoms. This could explain the late healthcare‐seeking and hospitalization.^22^ Physicians should consider early treatment and follow‐up of patients with influenza A/H1N1 to lower its mortality rate.

The high percentage of chronic conditions identified in this study among patients who died of influenza was also reported by others. Previous studies indicated that seasonal influenza infection is aggravated by existing chronic illness, with diabetes as the most frequently reported chronic condition among patients with severe influenza infection.^23^ This necessitates the early detection and treatment with antivirals in those patients.

This study proved that pneumonia as a complication was significantly higher in patients who died from influenza than those who discharged alive a finding reported from other studies.^6^, ^19^ This could be related to the high possibility of lung affection in A/H1N1 and should be considered in diagnosis and treatment of hospitalized patients with acute respiratory infection.

### 4.1 Study limitations

Data were available for four years which may not give an accurate depiction of influenza activity, epidemiology, and mortality in Egypt. We were unable to estimate influenza burden in Egypt because proportion of non‐participating healthcare facilities in the surveillance is unavailable. Data on chronic conditions were available only for patients died from influenza leading to inaccuracy in chronic diseases analysis as a risk for mortality, in addition to the unavailability of vaccination data on patients’ influenza vaccination status. Surveillance sensitivity and specificity were not tested, and application of the standard WHO case definition may be inconsistent due to large number participating of health facilities. Physicians tend to diagnose the more severe cases which may lead to underreporting and underestimation of the ARI problem in Egypt.

## 5 CONCLUSION

The National Egyptian Surveillance succeeded to accurately describe ARIs and influenza epidemiology in Egypt over time. Surveillance identified ARI as one of the leading causes of disease and influenza as a considerable cause of morbidity and mortality in Egypt. Influenza viruses are circulating in Egypt all over the year; however, activity differs by season, year, region, and subtype with patterns similar to those of the EMR and Northern Hemisphere. During the 2016‐2019, A/H1N1 caused more severe disease than H3N2 or B in hospitalized patients. Influenza severity and mortality are aggravated in winter, old age and with underlying medical conditions. Surveillance with strain‐specific laboratory testing can be useful, and annual assessment of associated severity might be important to guide influenza prevention and control strategies including vaccination and treatment. Elderly and persons with comorbidities should be specifically targeted for vaccination and early antiviral treatment to reduce the risk of influenza‐related complications. Further studies are needed to accurately describe the burden of epidemic‐prone acute ARI and influenza in Egypt to help developing evidence‐based disease prevention and control strategies.

## CONFLICT OF INTERESTS

None declared.

## AUTHOR CONTRIBUTIONS

**Manal Fahim:** Conceptualization (equal); Data curation (equal); Formal analysis (equal); Investigation (equal); Methodology (equal); Supervision (equal); Writing‐original draft (equal). **Basma AbdElGawad:** Investigation (equal); Methodology (equal); Writing‐review & editing (equal). **Hossam Hassan:** Investigation (equal); Methodology (equal); Validation (equal). **Amel Naguib:** Investigation (equal); Methodology (equal); Project administration (equal). **El Sabah Ahmed:** Investigation (equal); Software (equal); Validation (equal). **Hanaaa Abo ElSoud:** Project administration (equal); Supervision (equal); Writing‐review & editing (equal). **Salma Afifi:** Validation (equal); Writing‐original draft (equal); Writing‐review & editing (equal). **Amira Mohsen:** Project administration (equal); Supervision (equal); Writing‐review & editing (equal).

## ETHICAL APPROVAL

This study used the national acute respiratory surveillance data which is a public health activity organized by the Ministry of Health and Population in Egypt and has standing authorization from the National Ethical Committee. All personal identifiers were excluded.

### PEER REVIEW

The peer review history for this article is available at https://publons.com/publon/10.1111/irv.12867.

## Data Availability

The data that support the findings of this study are available from Egypt Ministry of Health and Population but restrictions apply to the availability of these data, which were used under license for the current study, and so are not publicly available. Data are however available from the Ministry of Health and Population upon reasonable request.
